# Isolated Cervical Rib Fracture: A Rare Etiology of Thoracic Outlet Syndrome

**DOI:** 10.1155/2011/163792

**Published:** 2011-10-20

**Authors:** Rayees Ahmad Dar, Sabiya Hamid Wani, Majid Mushtaque

**Affiliations:** ^1^Department of General and Minimal Invasive Surgery, Sher-i-Kashmir Institute of Medical Sciences, Soura, 190011 Srinagar, J&K, India; ^2^Department of General Surgery, Government Medical College, Srinagar, J&K, India; ^3^Department of Cardiovascular and Thoracic Surgery, Sher-I-Kashmir Institute of Medical Sciences, Soura, 190011 Srinagar, J&K, India

## Abstract

Isolated fracture of a cervical rib is a very rare entity and usually presents as a painless swelling or as thoracic outlet syndrome. We describe a case of a 45-year-old woman with history of fall two months back. She presented with symptoms of neurogenic thoracic outlet syndrome for one month. Isolated left cervical rib fracture was documented on X-ray cervical spine. Her fractured cervical rib was resected through a supraclavicular approach, and symptoms resolved completely in the postoperative period.

## 1. Introduction

By definition a cervical rib is an extra rib arising from the cervical vertebrae. It is usually associated with the 7th cervical vertebra. Anatomically, it is an enlarged costal element of the appropriate vertebra, causing the subclavian artery and vein and brachial plexus to arch over it. They are usually asymptomatic but may present with illdefined neurological or vascular symptoms, commonly referred to as the thoracic outlet syndrome (TOS) [1]. Cervical ribs typically produce root irritation or compression of the lower trunks of the brachial plexus resulting in pain or neurological loss in the hand. Cervical rib fracture due to neck trauma is an extremely rare cause of TOS [2]. Because of its position and the absence of muscle attachments, the only possible mechanism for a fracture of cervical rib is direct trauma. A case of isolated fracture of a cervical rib in a middle-aged woman is presented. The mechanism of injury was blunt trauma due to fall. She was not aware of the presence of a cervical rib. 

## 2. Case

**Figure 1:**
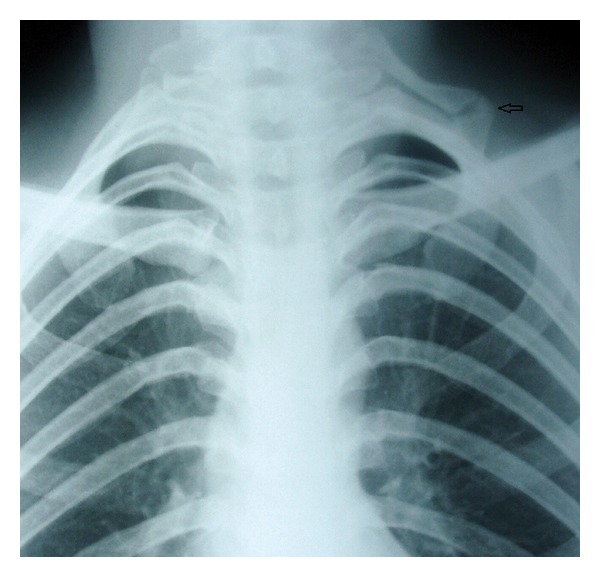
X-ray of chest and cervical spine (PA view) showing left cervical rib with fracture (arrow).

A 45-year-old woman, normotensive, nondiabetic, presented at the outpatient clinic of our institute. Her chief complaints were progressive pain, numbness, and tingling along the inner surface of her left forearm and lateral aspect of the fifth finger for one month. She had a history of fall (on her left shoulder) from about three meters two months back. At that time she was admitted on short stay basis and after certain investigations she was discharged home. She had no other complaints and no history suggestive of tuberculosis. General physical examination was normal in all aspects. There was tenderness in the left supraclavicular region. Left upper limb was warm, nontender, and distal pulses were present. Reflexes were intact. Her motor functions were normal. However, sensations were relatively diminished along medial aspect of left forearm and hand. A plain X-ray of cervical spine showed a left cervical rib which was fractured (Figure 1). Electrophysiologic study suggested the presence of left lower brachial plexus neuropathy. It was decided to remove fractured cervical rib. The patient was admitted, and cervical rib was excised along with first rib through supraclavicular approach. The patient made an uneventful recovery and was discharged on the 5th postoperative day. Her symptoms resolved completely in the postoperative period.

## 3. Discussion

According to Schein et al. [3], a cervical rib is present in 0.5–0.7% of the population and appears more commonly in females than males, in a ratio of 2 : 1. The condition is bilateral in about two-thirds of cases [4], but often the two sides are asymmetrical. Although a cervical rib is a congenital anomaly, it is most often an incidental finding causing no problems. When symptoms do appear they are usually initiated by sagging of the shoulder girdle, thus occurring mainly after the onset of middle age. The main manifestations are either neurological or vascular or both. Sensory function is first disturbed (paraesthesiae, pain, and clumsiness) and later motor function. Signs are most often found in the ulnar nerve distribution, but other nerves may also be involved. Vascular complications usually follow a well-known course. Halstead (as cited by Connell et al. [4]) was the first to report that when an artery is subjected to incomplete pressure an aneurysm develops distal to the point of pressure. This is due to changes in the turbulence of the blood flow. While this aneurysm develops, thrombus formation may occur, which may lead either to multiple small emboli causing eventual gangrene of the distal parts or to eventual total blockage of the subclavian artery. 

Isolated cervical rib fracture due to neck trauma is an extremely rare cause of TOS. As already mentioned, because of its position and the absence of muscle attachments, it is postulated that the only possible mechanism for a fracture is direct trauma. If TOS does develop, the rib should be excised to relieve the stretching of the nerves and artery. Surgery for neurogenic TOS in patients with cervical ribs should include both cervical and first rib resection. The failure rate for cervical rib resection without first rib resection in the work-related group was 75%. In contrast, when both cervical and first ribs were resected, the failure rate in the work-related group fell to 25% [5]. Connell et al. believe that, if secondary vascular changes are present, one should remove the affected part of the artery and replace it with a Teflon or vein graft. It is unnecessary to remove the whole rib, and one can leave about 1.5 cm of the proximal part. The periosteum should also be removed to prevent regeneration. The following conditions should be kept in mind in the differential diagnosis of TOS: (i) protruding intervertebral disc in the lower cervical spine; (ii) a lung tumor (mainly of the lung apex) infiltrating the neurovascular structures; (iii) carpal tunnel syndrome; (iv) ulnar nerve compression at the elbow.

## 4. Conclusion

The first problem in our case was that an isolated fracture of a cervical rib is extremely rare, and very few cases have been reported so far. The second problem arose as to why the patient developed signs of a thoracic outlet syndrome one month after her original injury. It may be that movement at the fracture site caused increased pressure on the neurovascular structures. This was greatly enhanced by edema and general inflammatory changes due to continuous friction at the fracture site. Surgery for neurogenic TOS in patients with cervical ribs should include both cervical and first rib resection.

## References

[1] Rayan GM (1988). Lower trunk brachial plexus compression neuropathy due to cervical rib in young athletes. *American Journal of Sports Medicine*.

[2] Sabapathy SR, Venkatramani H, Bhardwaj P (2010). Pseudarthrosis of cervical rib: an unusual cause of thoracic outlet syndrome. *Journal of Hand Surgery*.

[3] Schein CJ, Haimovici H, Young H (1956). Arterial thrombosis associated with cervical ribs: surgical considerations. *Surgery*.

[4] Connell JL, Doyle JC, Gurry JF (1980). The vascular complications of cervical rib. *Australian and New Zealand Journal of Surgery*.

[5] Sanders RJ, Hammond SL (2002). Management of cervical ribs and anomalous first ribs causing neurogenic thoracic outlet syndrome. *Journal of Vascular Surgery*.

